# Effectiveness of Vertical versus Horizontal Plyometric Training on Stretch-Shortening Cycle Performance Enhancement in Adolescent Soccer Players

**DOI:** 10.3390/healthcare11111615

**Published:** 2023-06-01

**Authors:** Cem Kurt, Umut Canli, Sadullah Erdal Erdaş, Luca Poli, Roberto Carvutto, Stefania Cataldi, Francesco Fischetti, Gianpiero Greco

**Affiliations:** 1 Faculty of Kırkpınar Sport Sciences, Trakya University, Edirne 22030, Turkey; cemkurt@trakya.edu.tr (C.K.); serdalerdas@gmail.com (S.E.E.); 2 Faculty of Sport Science, Tekirdağ Namık Kemal University, Tekirdağ 59030, Turkey; ucanli@nku.edu.tr; 3 Department of Translational Biomedicine and Neuroscience (DiBraiN), University of Study of Bari, 70124 Bari, Italy; luca.poli@uniba.it (L.P.); roberto.carvutto@uniba.it (R.C.); francesco.fischetti@uniba.it (F.F.); gianpiero.greco@uniba.it (G.G.)

**Keywords:** stretch-shortening cycle, adolescent players, soccer, plyometric

## Abstract

Plyometric exercise is a major tool for improving explosive actions. This study aimed to compare the efficacy of a vertical and horizontal plyometric training program on stretch-shortening performance variables in adolescent soccer players. Thirty-two male soccer players (aged 12.09 ± 0.89 years, with soccer experience 5.37 ± 1.58 years) were divided into horizontal plyometric, vertical plyometric, or control groups. The horizontal and vertical plyometric groups participated in a 6-week training program that was performed twice per week, with a 48-h interval, in conjunction with regular soccer training. The control group participated only in regular soccer training. Vertical jump height, reactive strength index, leg stiffness, ground contact time, standing long jump distance, agility, and 10 and 20 m sprint performances were tested as stretch-shortening performance variables of the participants. Stretch-shortening performance variables were assessed before and after completion of the training program. The results showed that either horizontal or vertical plyometric training had no effect on VJH, RSI, GCT, or Kleg performance (F = 2.14, 1.32, 0.66, 1.03; *p* > 0.05). Furthermore, there was no effect on SLJ, 10 m sprint, 20 m sprint, or agility performance (F = 2.06, 0.14, 0.06, 0.27; *p* > 0.05). A 6-week horizontal or vertical plyometric intervention was found to be insufficient to elicit stretch-shortening performance enhancement in adolescent male soccer players. Although there was no performance change in any group, it was observed that the players enjoyed plyometric training. Therefore, coaches could safely use plyometric exercises to design enjoyable training programs.

## 1. Introduction

Soccer, as a typical intermittent-type sport, incorporates a variety of explosive ballistic motions such as sprinting, kicking, jumping, accelerations and decelerations, tackling, direction changes, and turning [1]. Sprinting, jumping, and changing direction, as well as speed and acceleration, are important determinants of soccer success [2,3]. Therefore, the development of speed, jumping performance, and agility through specific training interventions should be emphasized in a soccer training program [4].

Plyometric training is widely utilized to increase physical performance in a variety of sports that involve sprinting, jumping, and changing direction [5,6,7,8]. It mainly consists of exercises that employ the stretch-shortening cycle (SSC), where a lengthening (eccentric) action is quickly followed by a shortening (concentric) movement [9,10]. Almost all explosive actions in soccer (sprinting, jumping, and changing direction) involve a stretch-shortening cycle (SSC) [11]. The effective use of the SSC is related to the contributions of different mechanisms, such as the accumulation of elastic energy [9], pre-load [12], increase of the time to muscle activation [13], muscle history dependence (force enhancement) [14], stretch reflexes [15], and muscle–tendon interactions [16] that facilitate greater mechanical work production in subsequent concentric muscle actions [17,18].

Plyometric training appears to have positive effects on physical abilities such as strength, power, explosiveness, and even endurance performance [19,20], as well as athletic performance measures such as sprint time, direction-changing skills, and jump performance [19,21,22,23]. Additionally, there is evidence that including plyometric training in a regular soccer training program can improve players’ speed [24,25,26], vertical jumping ability [27], and agility [28]. In light of this, recent research [1,7,29,30] has concentrated on the effectiveness of various plyometric training methods (vertical or horizontal) to improve the explosive characteristics in soccer players. However, adequate data about the best plyometric training program (vertical vs. horizontal) are lacking [4].

Only a small amount of research, however, has examined the efficacy of horizontal versus vertical plyometric training in young soccer players. Loturco et al. [31], in particular, evaluated the effects of horizontal and vertical plyometric training in young soccer players. According to the authors, horizontal plyometric training can cause greater adaptations to acceleration (up to 10 m), whereas vertical plyometric training appears to have more favorable effects for longer sprint distances (i.e., 10 to 20 m). Ramirez Campillo et al. [3] also compared the efficacy of horizontal and vertical plyometric training on different explosive indices in young (10–14-year-old) soccer players, reporting that both training routines may induce similar or slightly different neuromuscular adaptations during the in-season period. Meylan and Malatesta [26] found that an 8-week plyometric training program that included vertical and horizontal plyometric exercises improved 10 m sprint, agility, and jump height after rebound in early prepubertal soccer players (aged 13 years), while the control group showed no improvement. According to Manouras et al. [4], neither vertical horizontal plyometric nor vertical plyometric exercises improved standing long jump (SLJ) distance, vertical jump height (VJH), 10–30 m sprint, or agility performance in young male soccer players (aged 19–20 years).

Assessing the impact of horizontally or vertically oriented plyometrics on different phases of the sprint (e.g., acceleration and top-speed) might help coaches choose the training method that best matches the athletes’ traits and shortcomings. It is critical to emphasize that plyometrics are a field-based time-saving method for improving soccer players’ performance [31]. Despite the fact that plyometric exercise is an important tool for improving explosive actions (sprint, agility, and jump), there is no agreement on the efficacy of plyometric exercises that use different planes or which is more effective (vertical or horizontal) in enhancing performance, particularly in adolescent soccer players. As a result, the purpose of this study was to compare the effectiveness of vertical and horizontal plyometric exercises on SSC performance enhancement in adolescent soccer players, including the VJH, (reactive strength index) RSI, leg stiffness (Kleg), ground contact time (GCT), SLJ distance, 10 and 20 m sprint, and agility performance. We hypothesized that: (a) vertical plyometric (VP) exercises would improve VJH, RSI, Kleg, and GCT more than horizontal plyometric (HP) exercises; (b) HP exercises would improve SLJ and 10 and 20 m sprint time more than VP exercises; (c) both VP and HP exercises would improve VJH, RSI, Kleg, GCT, SLJ, and 10–20 m sprint time more than the control group.

## 2. Materials and Methods

### 2.1. Participants

Thirty-two adolescent male soccer players were recruited for this study. All players were members of the same soccer club competing in the U11–U13 leagues of the Edirne province of Turkey. Players regularly participated in soccer training twice weekly (average 90 min) and one soccer match every weekend. None of the participants had any prior experience with plyometric or strength training. Before the study, both participants and their parents or guardians were informed about the research protocol and the potential risks and benefits of participating in the study; they signed an informed consent form. The study was carried out in accordance with the Helsinki Declaration and was approved by Trakya University’s Medical Faculty’s Medical Ethics Committee (TÜTF-BAEK 2021/322). The following criteria were required for inclusion: a soccer player in leagues U11–U13, a minimum of 5 years of experience in soccer, a soccer license for the 2021–2022 seasons, being healthy based on a medical examination for having a soccer license, and participation in organized soccer training for at least 3 months without any detraining sessions. Subjects with unresolved musculoskeletal disorders, reconstructive lower extremity surgery in the past 2 years, and a history of ankle, knee, or spine pathology in the 3 months were excluded. The participants were instructed to maintain their sleep and dietary habits during the study and to avoid exhausting physical activity for 48 h prior to testing.

### 2.2. Procedures

**Figure 1:**
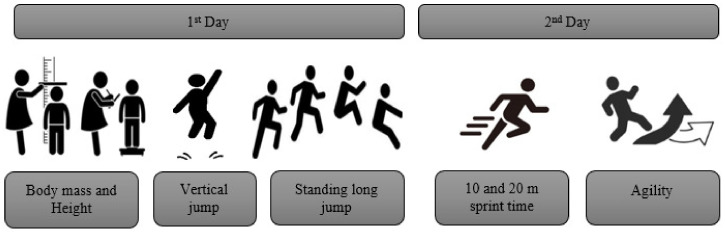
Study design overview.

Pre-and post-tests were performed over 2 days by the same researchers (See Figure 1 below). On the first day of the pre-test, the weekly training duration, soccer experience (years), body mass, height, VJH, and SLJ of the participants were determined. Body mass and height were measured to the nearest 0.1 cm and 0.1 kg, respectively, using a digital scale (SECA-769 by Seca, Hamburg, Germany). On the same day, the RSI, Kleg, and GCT of the participants were recorded automatically based on VJH using the Myotest Pro system accelerometer. On the second day of the pre-test, 10–20 m sprint time and agility performance were measured. The VJH, SLJ, RSI, Kleg, and GCT were measured on the first day (at 11 AM–2 PM) of the pre-and post-test in a sports hall with polyurethane ground. The agility performance and 10–20 sprint time were measured on the second day (7 PM–8.30 PM) pre-and post-test on the artificial grass soccer field. Using the analysis of the variance hypothesis test, three homogenous groups were constructed based on the participants’ pre-test values for 35 players. These groups were described as the horizontal plyometric training group (*n* = 12, HPG), the vertical plyometric training group (*n* = 12, VPG), and the control group (*n* = 11, CG). However, during the study, one player from each group dropped out and 32 completed the study: HPG, *n* = 11; VPG, *n* = 11; CG, *n* = 10.

### 2.3. Measurement

#### 2.3.1. Warm-Up Session

Each test session and plyometric training program began with a warm-up that included 10 min of self-paced jogging on the test field (similar to what happens before each soccer practice or match) and 5 min of dynamic stretching exercises that included a front kick with hand reach, side kick, back kick, butt kicks, high knee skipping, and walking lunge. Each exercise was repeated twice on a 10 m line. Participants were told to complete dynamic stretching exercises as quickly as possible.

#### 2.3.2. Reactive Jump Test

The VJH, RSI, GCT, and Kleg were all measured using a Myotest Pro system accelerometer (Myotest SA, Sion, Switzerland). Myotest is a valid and reliable method for the assessment of vertical jump height. The Myotest device is attached to the proper Velcro elastic belt and the belt is secured at hip level on the left side of the body for accelerometer recordings during a vertical jump [32]. The Myotest Pro system provides several performance tests, including half squats (HS), reactive jump tests, countermovement jumps (CMJ), and squat jumps, to evaluate an athlete’s performance (SJ). After recording the subjects’ body mass on it, the device was attached to the belt and fixed vertically. When the acoustic signal was activated, the subjects were instructed to hop in place five times as high as they could while lowering their GCT, with minimal knee flexion and maximum jump height. The participants were instructed to return to a vertical standing position after the final jump and await the final acoustic signal. The reactive jump test was completed after the final signal. The RSI, Kleg, GCT, and VJH were calculated automatically by the device. The procedure was repeated twice, with a three-minute break between each time. The best score from the two treatments was used in the statistical analysis [33].

#### 2.3.3. Standing Long Jump

To evaluate horizontal jump performance, the SLJ test was used. The participant was told to take a strong start and jump as far as possible from behind the starting line. The participant had to land on their feet and keep their balance. The distance between the take-off line and the point on the ground where the back of the heel closest to the take-off line landed was measured. Participants were encouraged to perform a maximum-effort SLJ [34] during the tests. Participants completed two maximal trials with a 45 s rest period after two familiarization trials, with the best performance used for statistical analysis.

#### 2.3.4. 10 and 20 m Sprint Test

The participants’ 10 and 20 m sprint times were recorded using a photocell gate (Newtest Powertimer 300-series, Oy, Helsinki, Finland). The test began with the subject standing and their front foot placed 0.2 m from the first photocell gate [35]. At first, each participant ran 2 × 10 m at 45 s intervals. The participants ran 2 × 20 m with a 1 min rest after a 3 min rest. During the sprint test, the participants verbally motivated themselves to perform at their best. The fastest 10 and 20 m sprint times were analyzed statistically.

#### 2.3.5. Pro-Agility Test

The propensity test was used to evaluate the participants’ agility performance. The subject started out in a neutral position. The subject was then instructed to sprint to either the dominant or non-dominant side first and touch a cone placed 5 m from the starting point. They were then told to run to the other side, touch the farthest cone at 10 m, and sprint 5 m to the finish line. The time was recorded using photocell gates (Newtest Powertimer 300-series, Oy, Finland). The subjects performed two trials on their dominant side, with the fastest time being analyzed. The subjects were given a 3 min break between trials.

### 2.4. Training Program

The VPG and HPG participated in a 6-week twice-weekly plyometric training program (12 training sessions in total). At the start of soccer practice, after 15 min of warm-up, vertical or horizontal plyometric exercises were performed. Because the team was competing, each training session lasted 90 min and included a 15 min warm-up, 20–30 min of plyometric exercises (horizontal or vertical), 40–45 min of technical and tactical soccer drills, and a 15 min cool-down. The VPG and HPG began their soccer training after completing their plyometric exercises. The CG did not participate in any special training other than team soccer practice. The total number of foot contacts increased from 60 to 90 with maximal effort during the plyometric intervention and all training sessions were supervised by the team’s coaches (See Table 1 below).

### 2.5. Statistical Analysis

All parameters were statistically analyzed using the SPSS 18 package program. The descriptive statistics (minimum and maximum values, mean, and standard deviation) of the participants were determined in terms of age, anthropometric structure, and sports life. The homogeneity of the data was assessed using kurtosis and skewness values. The participants’ body weight changes were determined using a paired sample *t*-test and a one-way analysis of variance (ANOVA) between groups. Furthermore, in repeated measures, the two-factor ANOVA test was used to determine the interaction of changes in the performance values of the participants (post-test/pre-test) at the group level (measurement group effect). The significance level was set at *p* < 0.05.

## 3. Results

The descriptive characteristics of the soccer players are given in Table 2. Two-way analysis of variance results in repeated measurements of vertical jump, RSI, GCT, Kleg, SLJ, 10 m sprint, 20 m sprint, and agility values of the HPG, VPG, and CG participants are presented in Table 3 and Table 4, respectively.

It was concluded that participating in either the horizontal or vertical plyometric training had no significant influence on VJH, RSI, GCT, or Kleg performance (post-test–pre-test difference) (F = 2.14, 1.32, 0.66, 1.03; *p* > 0.05).

It was discovered that participating in either the horizontal or vertical plyometric training groups had no significant influence on SLJ, 10 m sprint, 20 m sprint, or agility performance (post-test/pre-test difference, respectively: F = 2.06, 0.14, 0.06, 0.27; *p* > 0.05).

When the results of the participants’ in-group body mass changes were examined, it was determined that there were significant increases between the first and last test values in the HPG and CG (*p* < 0.05). The body mass changes in the VPG were not found to be significant (*p* > 0.05). Additionally, according to ANOVA test results for participants’ body mass changes, there were no statistically significant differences in body mass changes between the groups (*p* > 0.05).

## 4. Discussion

The aim of this study was to determine the effectiveness of vertical vs. horizontal plyometric exercises on SSC performance (VJH, SLJ, RSI, GCT, Kleg, agility, 10 and 20 m sprinting) enhancement in adolescent soccer players. The main finding was that participating in either horizontal or vertical plyometric training had no significant influence on VJH, SLJ, RSI, GCT, Kleg, 10 and 20 m sprinting, or agility performance. Based on this finding, none of the hypotheses of the study were able to be refuted.

Plyometric exercise responses are affected by several factors during childhood or adolescence, including the subjects’ age, gender, maturation status, body composition, physical fitness level, experience with plyometric drills, and plyometric training characteristics (intensity, number of foot contacts, frequency, resting time between sets, and recovery duration between training sessions) [36,37,38]. Although the majority of studies have looked into the effects of plyometric exercises on adolescent athletes’ physical performance [1,39,40,41,42,43,44], only a few have compared the efficacy of horizontal, vertical, or combined plyometric training in adolescents [3,4,31].

Manouras et al. [4] reported that an 8-week horizontal or vertical plyometric training performed 1 day per week in conjunction with conventional soccer training resulted in an improvement in agility, 30 m sprint performance, and VJ performance following training completion in both HPG and VPG, but only in the HPG. Loturco et al. [31] also reported that 11 sessions of vertical or horizontal plyometric training resulted in improved counter movement jump performance only in the VPG, improved horizontal jump distance in both groups, improved 10 m sprint time only in the HPG, and no improvement in the 20 m sprint time in any group. Participants in the preceding studies who were older (aged 18–20 years) than our subjects had a higher level of fitness and soccer experience. Ramrez-Campillo et al. [3] divided forty young soccer players aged 10–14 into four groups: control, vertical plyometric training, horizontal plyometric training, and combined horizontal and vertical training. Similarly, for 6 weeks, our study participants engaged in biweekly plyometric training with a 48-h interval. Volume (number of foot contacts) in their PT program changed from 80 contacts in the first week to 160 contacts in the last week. In comparison with the control group, the HPG improved only in horizontal counter movement jump performance and multiple bound tests, whereas the VPG improved in RSI, kicking velocity, 15–30 m sprint performance, balance, and change of direction speed. Ramirez-Campillo et al. [3] concluded that combining vertical and horizontal drills is more beneficial for improving performance. Disparities between Ramirez-Campillo et al. [3]’s study and our study could be attributed to PT programs, including plyometric drills and volume (foot contacts). During the study period, foot contact increased from 60 to 90 percent in our study (from the first to the last week). However, in the study by Ramirez-Campillo et al. [3], foot contacts changed from 80 to 160 during the study (from the first to the last week).

Although there is no one-size-fits-all physical therapy program, current evidence suggests that “a twice-week program for 8 to 10 weeks, beginning with 50–60 jumps a session and increasing weekly by 12–18 repetitions to a maximum of 90–190 results in the largest changes in running and jumping performance” [36]. Rubley et al. [41] recruited 16 adolescent soccer players (aged 13.4 ± 0.5 years) as the control and PT groups for their study. For 14 weeks, the PT group participated in a low-frequency and low-impact PT program in addition to regular soccer training; however, the control group only participated in regular soccer training. Both groups tested the vertical jump (VJ) height and kicking distance. The study highlighted that significant changes in the VJ and kicking distance performance did not occur until weeks 7 through 14 in the PT group. In contrast, Moore et al. [45] reported that decrement VJ decreased in the first 7 weeks of their study and found an enhancement of 7% by week 14. Based on the study by Rubley et al. [41] and Moore et al. [45], the 6-week PT performed in our study was insufficient to elicit performance enhancement and adaptation to PT exercise for reasons such as fatigue and neuromuscular maturation status of the adolescent soccer players.

The frequency of PT and recovery time between plyometric sessions may also explain the different results obtained across studies. PT programs can be performed 1–3 times a week, with 48–72 h recovery time [1,4,36,37,38]. In our study, soccer players participated in two plyometric training programs plus their soccer training, and they also played a league match every weekend. We were able to give 48 h recovery time between PT sessions. The participants trained every Tuesday and Thursday and played a match every Saturday. This situation might lead to fatigue; 48 h of resting between training was insufficient to recover. If we had applied one plyometric session in a week or 72 h rest time, we may have obtained different results.

**Figure 2:**
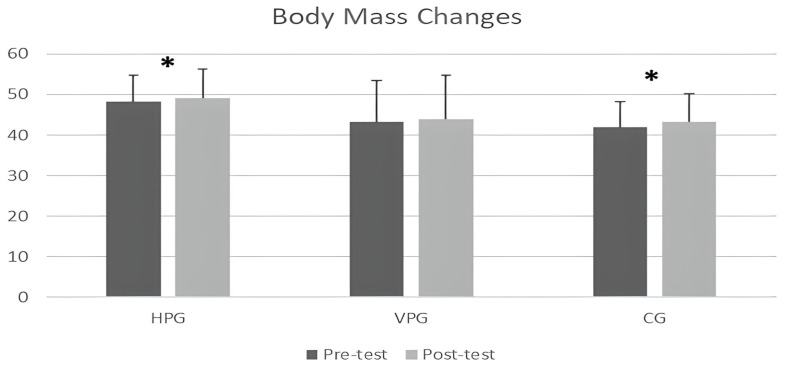
Paired sample *t*-test results for participants’ body mass changes. Notes: * *p* < 0.05; HPG—horizontal plyometric group, VPG—vertical plyometric group, CG—control group.

We also measured the participants’ body mass twice (pre to post), given that many performance parameters affect body mass fluctuations, such as VJH, sprint performance, agility performance, RSI, and Kleg [46]. Figure 2 shows that there were changes in body mass in the HPG and CG during the course of the study. However, the inter-group body mass changes in the HPG and CG did not lead to significant differences in body weight between the groups based on the post-tests. Therefore, the effectiveness of the plyometric training applied in this study might have concealed this body mass fluctuation in the HPG.

It is claimed that the participation of children or adolescents in plyometric exercises is a controversial topic [1,42]. However, some researchers argue that that well-designed plyometric exercises are effective for improving physical fitness in adults and pubertal children [4,36,41,47]. Considering the different results from the studies examined above, further investigations are needed to clarify the efficacy of vertical plyometric or horizontal plyometric exercises by using different plyometric training consisting of different intensities, volumes, durations, frequencies, and rest and recovery times in adolescent soccer players.

## 5. Conclusions

A 6-week horizontal or vertical plyometric intervention (twice a week, with a 48 h interval, 60–90 foot contacts in a session) was insufficient to elicit stretch-shortening performance enhancement, including VJH, SLJ, RSI, GCT, Kleg, agility, and 10 and 20 m sprint in adolescent male soccer players. The current study has some methodological limitations that need to be addressed. First, we did not calculate the statistical power of this study. Second, the soccer players were recruited from only one soccer club; therefore, when the study was replicated with a larger group, different results were obtained. Third, our findings were limited to one particular group of players. Further investigations with other age groups and competitive levels are necessary. Finally, although some authors have argued that peak height velocity (PHV) affects adolescents’ physical performance [39,42,48], we did not determine the PHV of our players. Although there was no performance change in any group, it has been observed that players enjoy plyometric training; therefore, coaches can safely use plyometric exercises to design enjoyable training programs.

## Figures and Tables

**Table 1:** Training load during the vertical plyometric and horizontal training programs.

Vertical Plyometric Training					Horizontal Plyometric Training			
Week	Exercises	Sets	Contacts	Total	Exercises	Sets	Contacts	Total
1–2	Vertical ankle jump	4	4	60	Horizontal obstacle jump	4	4	60
	Front obstacle jump	4	5		Heidens	4	5	
	Countermovement jump	4	6		Broad jump	4	6	
3–4	Vertical ankle jump	6	5	80	Kneeling jump	5	5	80
	Tuck jump	5	5		Heidens	6	5	
	Countermovement jump	5	5		Broad jump	5	5	
5–6	Foot fire	5	6	90	Kneeling to broad jump	5	6	90
	Pogo tuck jump	5	6		Diagonal jump	5	6	
	Squat jump with pause	5	6		Multiple diagonal jump	5	6	

Notes: Intensity: exercises were performed with maximal effort (intensity 100%); rest, 90 s between sets and 3 min between exercises. A 30 cm obstacle was used for the obstacle jump exercises.

**Table 2:** Descriptive statistics of the players.

Variables	Minimum	Maximum	Mean	SD
Age (years)	11.00	13.00	12.09	0.89
Body mass (kg)	23.90	64.20	44.59	8.04
Height (cm)	130.00	177.00	155.01	9.97
Weekly training (week/hours)	3.00	30.00	4.18	5.09
Sports experience (year)	2.50	9.00	5.37	1.58

Note: SD—standard deviation.

**Table 3:** Two-way analysis of variance results in repeated measurements of VJH, RSI, GCT, and Kleg values of the HPG, VPG, and CG participants.

Variables	Groups	Pre-Test	Post-Test	Group × Measurement		η_p_^2^	Bonferroni Post Hoc
		Mean ± SD	Mean ± SD	F	*p*		
**Vertical jump (cm)**	HPG	23.51 ± 4.06	23.34 ± 3.83	2.14	0.13	-	-
	VPG	27.60 ± 4.29	27.60 ± 4.47				
	CG	28.12 ± 4.86	25.89 ± 4.87				
**RSI (mm/ms)**	HPG	2.99 ± 0.67	3.11 ± 0.50	1.32	0.28	-	-
	VPG	3.55 ± 0.65	3.55 ± 0.66				
	CG	3.10 ± 0.78	3.37 ± 0.49				
**GCT (ms)**	HPG	146.81 ± 22.71	140.18 ± 21.22	0.66	0.52	-	-
	VPG	138.18 ± 39.07	134.36 ± 25.33				
	CG	150.90 ± 34.42	135.40 ± 19.19				
**K_leg_ (N/m)**	HPG	36.50 ± 14.78	40.10 ± 21.85	1.03	0.36	-	-
	VPG	33.44 ± 15.15	32.03 ± 11.79				
	CG	24.00 ± 11.78	29.17 ± 6.55				

Notes: HPG—horizontal plyometric group, VPG—vertical plyometric group, CG—control group, RSI—reactive strength index, GCT—ground contact time, Kleg—leg stiffness; η_p_^2^ = partial eta squared.

**Table 4:** Two-way analysis of variance results of t-repeated measurements of SLJ, 10 m sprint, 20 m sprint, and agility values of the HPG, VPG, and CG participants.

Variables	Groups	Pre-Test	Post-Test	Group × Measurement		η_p_^2^	Bonferroni Post Hoc
		Mean ± SD	Mean ± SD	F	*p*		
**Standing long jump (cm)**	HPG	150.18 ± 29.71	155.09 ± 32.12	2.06	0.14	-	-
	VPG	174.72 ± 22.95	172.27 ± 24.25				
	CG	169.10 ± 19.59	162.40 ± 15.67				
**10 m sprint (s)**	HPG	2.03 ± 0.21	1.97 ± 0.33	0.14	0.86	-	-
	VPG	1.93 ± 0.15	1.83 ± 0.13				
	CG	1.91 ± 0.11	1.84 ± 0.15				
**20 m sprint (s)**	HPG	3.69 ± 0.40	3.57 ± 0.47	0.06	0.93	-	-
	VPG	3.50 ± 0.31	3.41 ± 0.26				
	CG	3.53 ± 0.19	3.44 ± 0.23				
**Agility (s)**	HPG	5.63 ± 0.51	5.66 ± 0.56	0.27	0.76	-	-
	VPG	5.37 ± 0.39	5.39 ± 0.41				
	CG	5.50 ± 0.28	5.44 ± 0.50				

Notes: HPG—horizontal plyometric group, VPG—vertical plyometric group, CG—control group; η_p_^2^ = partial eta squared.

## Data Availability

Data are available for research purposes upon reasonable request to the first author.
